# Positron Emission Tomography-Computed Tomography (PET-CT) Diagnosis of Large-Vessel Giant Cell Arteritis Presenting as Pyrexia of Unknown Origin Without Classical Cranial Features: A Case Report

**DOI:** 10.7759/cureus.117046

**Published:** 2026-09-28

**Authors:** Aima Hameed, Siddhi A Gaunekar, Najat Haroun, Jyotsna Kakarla, Yasir Mehmood, Ajmal Raoof

**Affiliations:** 1 General Internal Medicine, Kettering General Hospital NHS Foundation Trust, Kettering, GBR; 2 General Medicine, Kettering General Hospital NHS Foundation Trust, Kettering, GBR; 3 Medicine, Kettering General Hospital NHS Foundation Trust, Kettering, GBR; 4 Internal Medicine, Kettering General Hospital NHS Foundation Trust, Kettering, GBR

**Keywords:** ana negative, anca negative, fdg pet-ct, giant cell arteritis (gca), large-vessel vasculitis and gca, pyrexia of unknown origin (puo)

## Abstract

Giant cell arteritis (GCA) is a systemic vasculitis affecting medium and large arteries and is classically associated with cranial manifestations such as headache, scalp tenderness, jaw claudication, and visual disturbance. However, large-vessel GCA may present with non-specific constitutional symptoms, making diagnosis difficult and delaying appropriate treatment. We report the case of a 58-year-old man with a history of hypertension, type 2 diabetes mellitus, osteoarthritis, and irritable bowel syndrome who presented with a 5-6-week history of intermittent fever, night sweats, anorexia, weight loss, mild headache, and predominantly lower-limb myalgia. Extensive investigations for infection, malignancy, and autoimmune disease were unrevealing. Microbiological investigations including blood and urine cultures, viral studies, acid-fast bacilli testing, and fungal markers were negative. Autoimmune screening, including antinuclear antibodies (ANA), antineutrophil cytoplasmic antibodies (ANCA), rheumatoid factor, and anti-cyclic citrullinated peptide antibodies (anti-CCP), was also negative. Computed tomography (CT) of the chest, abdomen, and pelvis showed no evidence of occult infection or malignancy.

Despite empirical antibiotic therapy, the patient remained symptomatic with persistently elevated inflammatory markers, including an erythrocyte sedimentation rate of 136 mm/hr and C-reactive protein of 196 mg/L. As part of the evaluation for pyrexia of unknown origin, fluorodeoxyglucose positron emission tomography-computed tomography (FDG PET-CT) was performed, which demonstrated increased uptake within the bilateral femoral, vertebral, and axillary arteries, consistent with large-vessel vasculitis. Following rheumatology assessment and specialist consultation, a clinical and radiological diagnosis of large-vessel GCA was made. High-dose oral prednisolone was initiated, resulting in rapid clinical and biochemical improvement, with marked improvement in inflammatory markers. A subsequent right temporal artery biopsy demonstrated histological features in keeping with GCA.

This case illustrates an atypical presentation of GCA as pyrexia of unknown origin without classic cranial or visual symptoms. Several important learning points emerge. Firstly, clinicians should maintain a high level of suspicion for large-vessel vasculitis in patients over 50 who present with persistent systemic inflammation and non-specific constitutional symptoms, specifically prolonged fever, weight loss, and myalgia, even in the absence of cranial manifestations. Secondly, the case demonstrates the diagnostic utility of FDG PET-CT in identifying large-vessel vascular inflammation when initial standard workup for infection, malignancy, and autoimmune disease is unrevealing. Thirdly, in selected patients with suspected large-vessel vasculitis, FDG PET-CT can provide an important diagnostic clue and help guide timely specialist management.

## Introduction

Giant cell arteritis (GCA) is a systemic vasculitis affecting medium and large arteries, most commonly occurring in individuals over the age of 50 [1]. It is classically associated with cranial manifestations, including new-onset temporal headache, scalp tenderness, jaw claudication, and visual disturbance [1,2]. Immediate recognition is essential because untreated disease can result in serious ischemic complications, including irreversible visual loss [1].

Although GCA is often conceptualized as temporal arteritis, it is increasingly understood as a heterogeneous disease with both cranial and extracranial phenotypes. Large-vessel GCA may involve the aorta and its major branches and can present with constitutional symptoms, polymyalgia rheumatica-like symptoms, limb symptoms, pyrexia of unknown origin, or isolated inflammatory marker elevation [2,3]. Compared with cranial GCA, large-vessel GCA is associated with fewer cranial manifestations, lower rates of positive temporal artery biopsy, and longer diagnostic delay [3]. The absence of typical cranial manifestations may reduce early clinical suspicion and contribute to diagnostic delay [3].

Fluorodeoxyglucose positron emission tomography-computed tomography (FDG PET-CT) has an important role in the evaluation of suspected large-vessel vasculitis and can identify metabolically active vascular inflammation when conventional investigations are inconclusive [4,5]. Contemporary European Alliance of Associations for Rheumatology (EULAR) recommendations consider ultrasound of the temporal and axillary arteries the first-line imaging modality in suspected GCA, with FDG PET-CT providing a complementary option when extracranial large-vessel involvement is suspected or further vascular assessment is required [4]. We present a case of large-vessel GCA in a 58-year-old man presenting with prolonged pyrexia of unknown origin, constitutional symptoms, and lower-limb myalgia, without classical cranial or visual features. The diagnosis was made clinically and radiologically following FDG PET-CT, which demonstrated large-vessel uptake involving the femoral, vertebral, and axillary arteries. A subsequent right temporal artery biopsy demonstrated histological features in keeping with GCA.

## Case presentation

A 58-year-old White man with a past medical history of hypertension, type 2 diabetes mellitus, osteoarthritis, and irritable bowel syndrome was admitted for investigation of pyrexia of unknown origin. He reported a 5-6-week history of intermittent fever, including a documented temperature of 38.2°C during a Medical Same Day Emergency Care (MSDEC) assessment and temperatures of 38.3°C on two occasions during the subsequent inpatient admission, accompanied by loss of appetite, night sweats, mild headache, and generalized muscle aches. His myalgia was most prominent in the lower limbs, particularly around the hips and thighs, and he described the symptoms as muscular rather than articular. He also reported unintentional weight loss of approximately 6.4 kg (14 pounds) since the onset of symptoms.

Before admission, he had been assessed through urgent care, the emergency department, and the Same Day Emergency Care pathway. He was initially treated empirically with a 10-day course of co-amoxiclav for presumed infection, but his symptoms persisted, and inflammatory markers remained markedly elevated, with pre-admission C-reactive protein (CRP) values of 213 mg/L and 188 mg/L documented on separate assessments. No pre-admission erythrocyte sedimentation rate (ESR) value was available. He denied localizing infective symptoms, including cough, sore throat, urinary symptoms, abdominal pain, bowel disturbance, or skin rash. There was no recent travel, recreational drug use, high-risk sexual history, or contact with unwell individuals. He had domestic pets, including one dog and four cats. He also reported tick exposure during woodland activity the previous year, for which he had received antibiotics at the time; Lyme serology was reportedly negative, and there had been no recent tick bite. Given the prolonged duration of fever, recurrent documented febrile episodes, and absence of a diagnosis despite repeated investigations, the presentation was considered consistent with pyrexia of unknown origin [6].

On examination, he appeared clinically well between febrile episodes. He was tachycardic, with an electrocardiogram demonstrating sinus tachycardia. Cardiovascular, respiratory, and abdominal examinations were otherwise unremarkable. There was no lymphadenopathy, rash, synovitis, or calf tenderness. Although he reported a mild non-specific headache, he did not describe jaw claudication, visual disturbance, scalp tenderness, or temporal artery tenderness. There were no neurological symptoms or features of meningism.

Initial investigations were guided by the need to assess for underlying infection, inflammation, or malignancy in the context of persistent systemic symptoms. Inflammatory markers were markedly raised, with ESR reaching 136 mm/hr and CRP reaching 196 mg/L. Additional laboratory tests were selected to investigate potential differential diagnoses, revealing hyperferritinemia, thrombocytosis, raised fibrinogen, elevated complement C3, and lactate dehydrogenase of 262 IU/L. Procalcitonin testing was performed to help distinguish between bacterial infection and non-infectious inflammatory causes, which was negative (Table 1).

**Table 1:** Biochemistry/general blood tests

Test	Result	Reference range	Flag
Hemoglobin	117 g/L	130-180 g/L	Low
White blood cells	10.6x10^9^/L	4-11x10^9^/L	Normal
Platelets	488x10^9^/L	150-450x10^9^/L	High
Neutrophils	8.5x10^9^/L	2-7.5x10^9^/L	High
Lymphocytes	1x10^9^/L	1.5-4.5x10^9^/L	Low
Monocytes	0.9x10^9^/L	0.2-0.8x10^9^/L	Normal
Eosinophils	0.2x10^9^/L	0-0.4x10^9^/L	Normal
Basophils	0.1x10^9^/L	0-0.1x10^9^/L	Normal
C-reactive protein	196 mg/L	<5 mg/L	High
Erythrocyte sedimentation rate	136 mm/hr	2-10 mm/hr	High
Sodium	137 mmol/L	133-146 mmol/L	Normal
Potassium	4.6 mmol/L	3.5-5.3 mmol/L	Normal
Urea	4.2 mmol/L	2.15-7.1 mmol/L	Normal
Creatinine	59 µmol/L	59-104 µmol/L	Normal
Estimated glomerular filtration rate	>90 mL/min/1.73 m^2^	>59 mL/min/1.73 m^2^	Normal
Creatine kinase	95 IU/L	40-320 IU/L	Normal
Thyroid‑stimulating hormone	1.09 mIU/L	0.27-4.20 mIU/L	Normal
Lipid profile: cholesterol	3.6 mmol/L	<5.2 mmol/L	Normal
Lipid profile: triglycerides	1 mmol/L	0.8-2.3 mmol/L	Normal
Ferritin	1158 ng/mL	43-518 ng/mL	High
Fibrinogen	8.4 g/L	1.8-3.5 g/L	High
Angiotensin‑converting enzyme	27 U/L	20-70 U/L	Normal
Lactate dehydrogenase	262 IU/L	<225 IU/L	High
Procalcitonin	0.14 ng/mL	<0.25 ng/mL	Normal

An extensive infectious workup did not identify a source. Blood cultures, urine cultures, influenza and SARS-CoV-2 testing, Epstein-Barr virus and cytomegalovirus screens, acid-fast bacilli testing, and fungal markers were negative. QuantiFERON-TB Gold testing was indeterminate and therefore did not provide an interpretable result for tuberculosis screening. Urine microscopy showed no evidence of infection (Table 2).

**Table 2:** Infectious disease serology and microbiology *Borrelia *IgG/IgM: *Borrelia *immunoglobulin G and immunoglobulin M antibodies; *Treponema *EIA: *Treponema *enzyme immunoassay; AFB: acid-fast bacilli; QuantiFERON‑TB Gold: Interferon Gamma Release Assay for Tuberculosis; Galactomannan antigen: *Aspergillus *galactomannan antigen; B‑glucan: fungal beta-glucan assay; PCR: polymerase chain reaction

Test	Result	Flag
*Borrelia *IgG/IgM	Negative	Negative
*Treponema *EIA	Negative	Negative
AFB	Negative	Negative
QuantiFERON-TB Gold	Indeterminate	Indeterminate
Galactomannan antigen	0.048	Negative
B-glucan	31 pg/ml	Negative
Blood cultures	Negative	Negative
Influenza PCR	Negative	Negative
COVID PCR	Negative	Negative
Respiratory PCR panel	Negative	Negative

Autoimmune testing was also unrevealing. Antinuclear antibodies, antineutrophil cytoplasmic antibodies, rheumatoid factor, and anti-cyclic citrullinated peptide antibodies were negative (Table 3). Extractable nuclear antigen and anti-double-stranded DNA testing were requested following rheumatology advice but were not processed by the laboratory because antinuclear antibodies were repeatedly negative. Tumor marker testing was also performed and was negative (Table 4).

**Table 3:** Immunology/autoimmune/immunoglobulins ANA: antinuclear antibody; ANCA: antineutrophil cytoplasmic antibodies; anti‑CCP: anti-cyclic citrullinated peptide antibodies; RF: rheumatoid factor; ASO: anti-streptolysin O antibody; C3: complement component 3; C4: complement component 4; EP: protein electrophoresis; IgG: immunoglobulin G; IgA: immunoglobulin A; IgM: immunoglobulin M; IgG4: immunoglobulin G subclass 4

Test	Result	Reference range	Flag
ANA	Negative	-	Normal
ANCA	Negative	-	Normal
Anti-CCP	0.8 U/mL	<7 U/mL	Normal
RF	1.9 IU/mL	<3.5 IU/mL	Normal
ASO	<200 IU/ml	0-200 IU/ml	Normal
C3	2.13 g/L	0.90-1.80 g/L	High
C4	0.39 g/L	0.14-0.54 g/L	Normal
EP	No paraprotein detected	-	Normal
IgG	11.28 g/L	6-16 g/L	Normal
IgA	4.01 g/L	0.80-4.00 g/L	High
IgM	0.89 g/L	0.50-2.00 g/L	Normal
IgG4	0.24 g/L	0.01-1.30 g/L	Normal

**Table 4:** Tumor markers PSA: prostate‑specific antigen; CEA: carcinoembryonic antigen; CA 19‑9: carbohydrate antigen 19‑9; AFP: alpha‑fetoprotein

Test	Result	Reference range	Flag
PSA	0.73 ug/L	<3.50 ug/L	Normal
CEA	<1 UG/L	0-4 UG/L	Normal
CA 19-9	11 U/mL	0-34 U/mL	Normal
AFP	<2 KU/L	<6 KU/L	Normal

Cross-sectional imaging did not identify an alternative diagnosis. Computed tomography (CT) of the chest, abdomen, and pelvis showed no evidence of occult infection, collection, significant lymphadenopathy, malignancy, or destructive bone lesion (Figure 1). CT of the head demonstrated no acute intracranial pathology and showed only chronic ischemic changes. Transthoracic echocardiography was limited by image quality but did not demonstrate obvious valvular abnormalities or large mobile masses, although infective endocarditis could not be excluded by transthoracic imaging alone.

**Figure 1:**
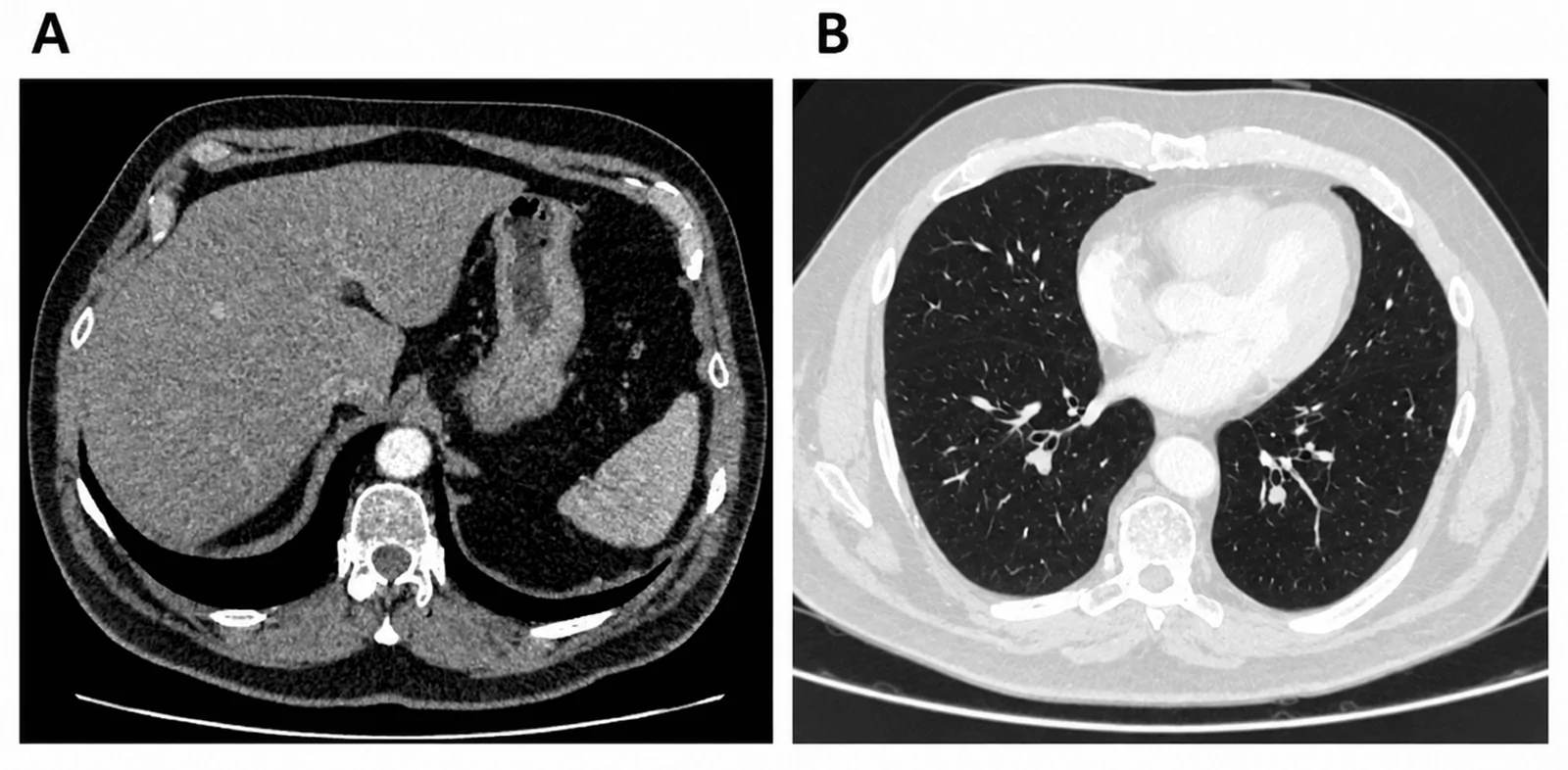
Representative CT images from the initial cross-sectional imaging Representative axial CT images of the upper abdomen (A) and chest (B) obtained during the initial evaluation for pyrexia of unknown origin. No clear infective, malignant, or inflammatory source was identified on CT imaging. CT: computed tomography

Given the persistence of pyrexia and inflammatory markers despite negative initial investigations, infectious diseases advice was sought. The recommendation was to proceed with FDG PET-CT alongside further blood tests and swabs.

FDG PET-CT was performed from the vertex to the knees following the administration of 324 MBq FDG; blood glucose at the time of imaging was 8.3 mmol/L, with reference maximum standardized uptake value (SUVmax) values of 3.3 for the mediastinum and 3.8 for the liver. FDG PET-CT demonstrated diffuse FDG uptake within the bilateral femoral arteries extending to the knees, with SUVmax up to 9.4, greater than liver uptake. Diffuse uptake was also seen within the bilateral vertebral arteries, with SUVmax up to 4.9. Focal uptake posterior to the angle and ramus of the mandible bilaterally, with SUVmax up to 4.5, was considered possibly vascular in origin. Both axillary arteries demonstrated uptake with SUVmax up to 3.6 (Figures 2-3). Overall appearances were reported as concerning for vasculitis, with vascular and rheumatology review recommended.

**Figure 2:**
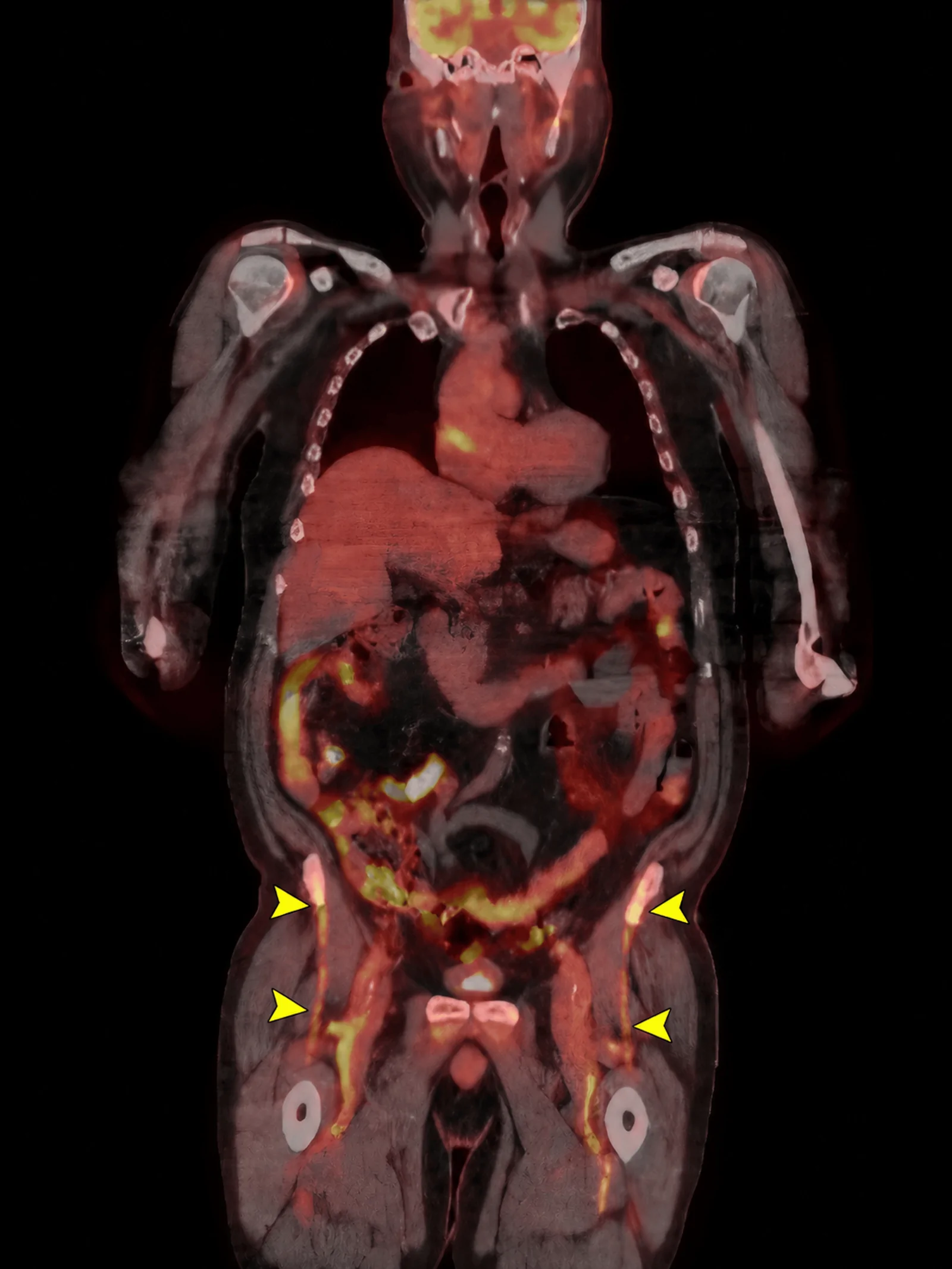
Representative FDG PET-CT findings suggestive of large-vessel vasculitis Coronal fused FDG PET-CT image demonstrating increased linear FDG uptake along the bilateral femoral arterial regions. Arrowheads indicate the regions of abnormal vascular uptake. Physiological bowel tracer uptake is also visible. FDG PET-CT: fluorodeoxyglucose positron emission tomography-computed tomography

**Figure 3:**
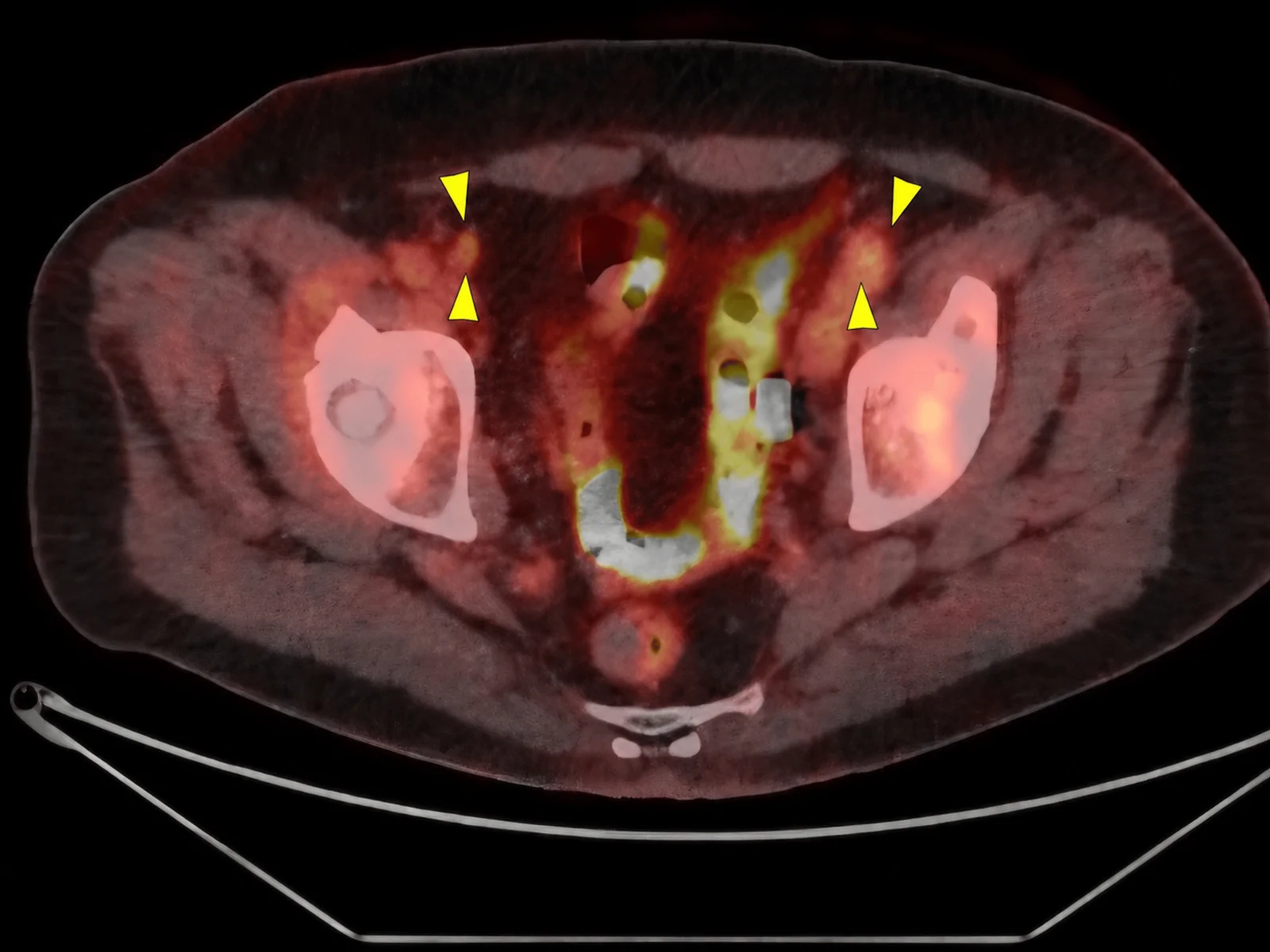
Representative FDG PET-CT findings suggestive of large-vessel vasculitis Axial fused FDG PET-CT image through the pelvis demonstrating increased FDG uptake in the femoral arterial regions. Arrowheads indicate the regions of abnormal vascular uptake. FDG PET-CT: fluorodeoxyglucose positron emission tomography-computed tomography

The patient was reviewed by rheumatology, and the PET-CT findings were felt to be in keeping with large-vessel vasculitis. Following specialist discussion with Addenbrooke's Hospital, Cambridge, further blood tests including immunoglobulins and IgG4 were requested. A clinical and radiological diagnosis of large-vessel GCA was made.

Temporal artery ultrasound was not performed during the inpatient admission. At the time of initial diagnosis and treatment, temporal artery biopsy had been requested but was still pending. During subsequent follow-up, a right temporal artery biopsy was performed and demonstrated chronic transmural inflammation with disruption of the internal elastic lamina and multinucleated giant cells, with features in keeping with giant cell arteritis.

He was prescribed oral prednisolone 60 mg once daily for four weeks, with a planned taper under rheumatology supervision. Lansoprazole was started for gastroprotection and calcium carbonate/colecalciferol for bone protection. Baseline HbA1c assessment was requested given his known type 2 diabetes mellitus and anticipated steroid exposure. *Pneumocystis jirovecii *pneumonia (PJP) prophylaxis was not initiated at the time of discharge.

Following corticosteroid therapy, the patient showed significant biochemical improvement. His ESR improved from 136 mm/hr in April to 28 mm/hr in June, and his CRP improved from 196 mg/L in April to less than 5 mg/L in June (Figure 4). Follow-up HbA1c in June was 68 mmol/mol.

**Figure 4:**
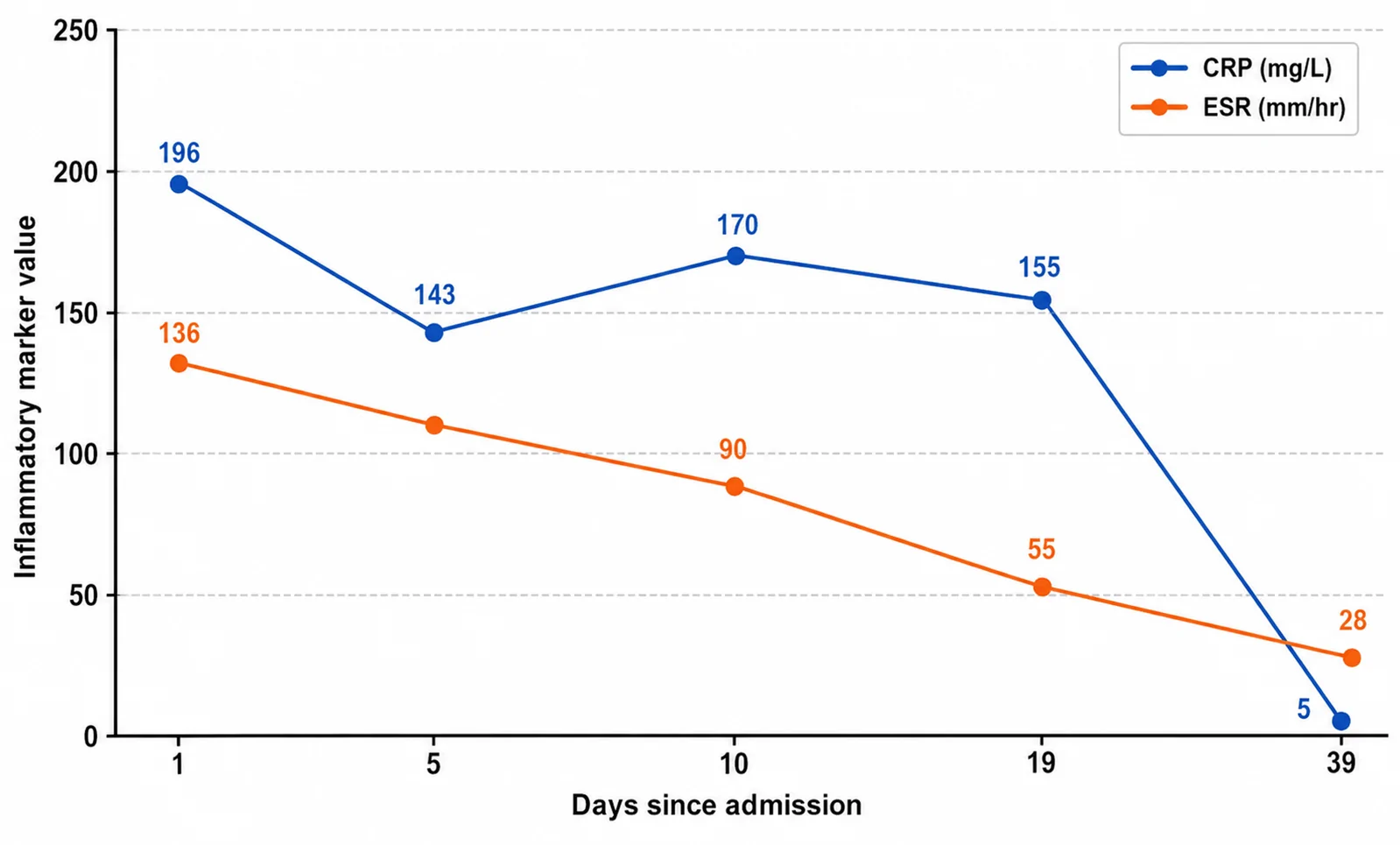
Trend in inflammatory markers during the clinical course Line graph showing serial inflammatory marker values by days since admission. The blue line represents CRP (mg/L), and the orange line represents ESR (mm/hr). Both markers remained elevated during the initial diagnostic period, followed by marked improvement after the initiation of corticosteroid therapy. CRP improved from 196 mg/L to 5 mg/L, while ESR improved from 136 mm/hr to 28 mm/hr. CRP: C-reactive protein; ESR: erythrocyte sedimentation rate

Upon discharge, he was scheduled for outpatient rheumatology follow-up for ongoing monitoring, including repeat inflammatory markers, assessment for steroid-related complications, and evaluation of symptom progression. At the time of discharge, arrangements had been made for temporal artery biopsy as part of the planned rheumatology follow-up, alongside further vascular imaging to assess disease extent and monitor for potential large-vessel complications. The multidisciplinary team, including rheumatology and vascular specialists, was involved in coordinating follow-up care and guiding glucocorticoid tapering according to clinical response.

The patient's clinical course from initial ED/MSDEC attendance to treatment response is summarized in Table 5.

**Table 5:** Timeline of the clinical course CRP: C-reactive protein; ESR: erythrocyte sedimentation rate; FDG PET-CT: fluorodeoxyglucose positron emission tomography-computed tomography; GCA: giant cell arteritis; ED: emergency department; MSDEC: Medical Same Day Emergency Care

Timepoint	Key event
Initial ED/MSDEC attendance	Fever of unclear source; commenced on 10-day co-amoxiclav
Repeat MSDEC review	Persistent fever, raised inflammatory markers, reduced appetite, weight loss, and shoulder/hip pain. CT of the chest/abdomen/pelvis, cultures, viral screen, and vasculitic screen requested
Subsequent MSDEC follow-up	CT of the chest/abdomen/pelvis, blood cultures, urine culture, and vasculitic screen were unrevealing. Night sweats and sinus tachycardia persisted
Further review after 5-6 weeks of symptoms	Ongoing night sweats, anorexia, mild headache, and hip/thigh myalgia; admitted for investigation of pyrexia of unknown origin
Inpatient workup	Extensive infectious, autoimmune, malignant, and radiological investigations did not identify an alternative cause
Infectious diseases input	Further investigations and FDG PET-CT advised
FDG PET-CT	Increased uptake in the bilateral femoral, vertebral, and axillary arteries; appearances concerning for large-vessel vasculitis
Specialist review	Rheumatology and tertiary center input supported a clinical and radiological diagnosis of large-vessel GCA. Temporal artery biopsy was recommended
Treatment and follow-up	High-dose prednisolone commenced, followed by clinical improvement and biochemical response
Subsequent follow-up	Right temporal artery biopsy demonstrated histological features in keeping with GCA

## Discussion

This report describes an atypical presentation of GCA, with persistent pyrexia of unknown origin occurring in the absence of classic cranial or visual symptoms. The patient's several-week history of intermittent fever, night sweats, anorexia, weight loss, mild headache, and lower-limb myalgia illustrates the unusual clinical pattern. Notably, the initial extensive workup failed to identify an infective, malignant, or systemic autoimmune etiology, and cross-sectional imaging was inconclusive. FDG PET-CT revealed vascular uptake suggestive of large-vessel vasculitis, supporting a clinical and radiological diagnosis and emphasizing the diagnostic challenge and clinical significance of this uncommon presentation.

GCA is classically associated with cranial symptoms such as temporal headache, scalp tenderness, jaw claudication, and visual disturbance [1,2]. However, large-vessel involvement is an important extracranial phenotype [2,3]. Patients with large-vessel GCA may present with constitutional symptoms, polymyalgia rheumatica-like pain, limb symptoms, or unexplained elevation of inflammatory markers [2,3]. A systematic review and meta-analysis of large-vessel GCA reported that this phenotype is associated with fewer cranial symptoms, delayed diagnosis, and lower rates of temporal artery biopsy positivity compared with cranial GCA [3]. This is clinically relevant because the absence of cranial features may reduce early diagnostic suspicion.

The patient had markedly elevated inflammatory markers, including ESR of 136 mm/hr and CRP of 196 mg/L, alongside thrombocytosis, hyperferritinemia, raised fibrinogen, and elevated complement C3. The elevated ferritin, C3, and mildly raised IgA were considered non-specific findings in the context of the systemic inflammatory response. Despite this significant inflammatory response, extensive microbiological investigations were unrevealing. Blood and urine cultures, viral testing, acid-fast bacilli testing, and fungal markers were negative, while QuantiFERON-TB Gold testing was indeterminate. Autoimmune screening, including antinuclear antibodies, antineutrophil cytoplasmic antibodies, rheumatoid factor, and anti-cyclic citrullinated peptide antibodies, was also negative. While these tests helped exclude alternative diagnoses, negative serology does not exclude GCA, as diagnosis relies on the overall clinical picture, inflammatory response, vascular imaging, and/or histological confirmation [1,4].

FDG PET-CT helped redirect the diagnostic workup toward large-vessel vasculitis in this case. An important practical consideration is the timing of imaging relative to glucocorticoid therapy, as vascular FDG uptake can decrease after treatment is started. Where feasible, FDG PET-CT should therefore be performed before, or very early after, glucocorticoid initiation, provided that imaging does not delay necessary treatment [4,5]. In this patient, PET-CT was performed before corticosteroid therapy was commenced. Conventional CT imaging of the chest, abdomen, and pelvis did not identify occult infection, malignancy, lymphadenopathy, or abscess formation. In contrast, PET-CT demonstrated diffuse FDG uptake within the femoral and vertebral arteries, with additional uptake in the axillary arteries and possible vascular uptake near the mandibular angles. The focal activity near the mandibular angles was considered possibly vascular in origin, although the patient had no corresponding cranial symptoms. These findings raised suspicion for large-vessel vasculitis and prompted rheumatology and tertiary center input. Importantly, this approach aligns with current guideline recommendations. The 2023 EULAR recommendations support the use of vascular imaging, including FDG PET-CT, as a diagnostic tool in suspected large-vessel vasculitis, particularly when extracranial involvement is suspected [4]. However, there are limitations to the use of PET-CT in this context. FDG uptake is non-specific and can be observed in several inflammatory, infective, and neoplastic processes, which may lead to false positives [5,6]. Additionally, PET-CT may not reliably distinguish active vasculitis from other causes of vascular inflammation or detect small-vessel involvement. PET-CT findings should therefore be interpreted in conjunction with the clinical presentation and laboratory data [4,5]. Even accounting for these limitations, this case underlines the value of a guideline-based diagnostic approach that incorporates PET-CT in selected patients with pyrexia of unknown origin and persistent systemic inflammation when large-vessel vasculitis is suspected and initial investigations are unrevealing [4-7].

The pattern of vascular involvement in this patient is notable. Large-vessel GCA commonly affects the aorta and its major branches, but extracranial involvement may be clinically silent or present with non-specific symptoms [2,3]. In this case, the patient's lower-limb myalgia and hip/thigh discomfort may have reflected systemic inflammation and were notable in the context of the femoral arterial uptake, although a direct causal relationship cannot be established and he did not report classic limb claudication. The absence of visual symptoms, jaw claudication, scalp tenderness, temporal artery tenderness, or temporal artery abnormalities contributed to the diagnostic challenge.

Previous reports have described GCA presenting as fever of unknown origin, including cases where PET-CT identified large-vessel vasculitis after conventional investigation was unrevealing [8,9]. Baymakova et al. described a case of GCA without cranial manifestations presenting as fever of unknown origin, in which FDG PET-CT demonstrated large-vessel uptake and supported early diagnosis and monitoring of treatment response [9]. The present case adds to this literature by demonstrating large-vessel GCA presenting with prolonged pyrexia of unknown origin and predominant lower-limb myalgia in the absence of classic cranial or visual symptoms.

Following specialist review, the patient was diagnosed with large-vessel GCA on clinical and radiological grounds and commenced on high-dose oral prednisolone. His subsequent clinical and biochemical response was significant, with CRP improving from 196 mg/L to less than 5 mg/L and ESR improving from 136 mm/hr to 28 mm/hr. This response was compatible with an inflammatory vasculitic process but was not diagnostic in isolation. Temporal artery biopsy had been requested but was still pending when treatment was initiated. During subsequent follow-up, right temporal artery biopsy demonstrated chronic transmural inflammation with disruption of the internal elastic lamina and multinucleated giant cells, with features in keeping with GCA. Although the biopsy was positive in this patient, temporal artery biopsy may be negative when extracranial vessels are predominantly involved [3,10]. Contemporary recommendations recognize the role of vascular imaging in suspected large-vessel vasculitis, particularly when extracranial involvement is suspected [1,4,10,11].

This case reinforces several important learning points. First, GCA should be considered in patients over 50 presenting with pyrexia of unknown origin and persistently elevated inflammatory markers, even in the absence of classic cranial symptoms [1-3]. Second, negative infectious and autoimmune investigations should prompt reconsideration of large-vessel vasculitis when systemic inflammation persists. Patients over 50 with persistent unexplained fever, markedly elevated inflammatory markers, and an unrevealing initial infectious, malignant, and autoimmune workup may warrant consideration of early vascular imaging, particularly when constitutional, polymyalgia-like, or limb symptoms are present [2-5]. Third, FDG PET-CT can be a valuable diagnostic tool in detecting extracranial vascular inflammation and guiding specialist management [4,5]. Early recognition is important to allow timely treatment and reduce the risk of complications [1,10].

## Conclusions

Large-vessel GCA may present as pyrexia of unknown origin without classical cranial or visual features. This case reinforces the need to maintain diagnostic suspicion for large-vessel vasculitis in patients over 50 with persistent constitutional symptoms and markedly elevated inflammatory markers despite an unrevealing infectious, malignant, and autoimmune workup.

FDG PET-CT can provide an important diagnostic clue by identifying metabolically active vascular inflammation when conventional imaging is unrevealing. In this case, the diagnosis was initially made on clinical and radiological grounds and was subsequently supported by temporal artery biopsy showing features in keeping with GCA. Early recognition and treatment are important to reduce the risk of complications and achieve clinical and biochemical improvement.
